# *Staphylococcus aureus* oleate hydratase produces ligands that activate host PPARα

**DOI:** 10.3389/fcimb.2024.1352810

**Published:** 2024-03-27

**Authors:** Christopher D. Radka, Matthew W. Frank, Tyler S. Simmons, Cydney N. Johnson, Jason W. Rosch, Charles O. Rock

**Affiliations:** ^1^ Department of Microbiology, Immunology, and Molecular Genetics, University of Kentucky, Lexington, KY, United States; ^2^ Department of Host Microbe Interactions, St. Jude Children’s Research Hospital, Memphis, TN, United States

**Keywords:** oleate hydratase, hydroxy fatty acid, macrophage, *Staphylococcus aureus*, peroxisome proliferator activated receptor alpha (PPARα), fatty acid oxidation (FAO), host-microbe interaction, signaling

## Abstract

Commensal gut bacteria use oleate hydratase to release a spectrum of hydroxylated fatty acids using host-derived unsaturated fatty acids. These compounds are thought to attenuate the immune response, but the underlying signaling mechanism(s) remain to be established. The pathogen *Staphylococcus aureus* also expresses an oleate hydratase and 10-hydroxyoctadecanoic acid (*h*18:0) is the most abundant oleate hydratase metabolite found at Staphylococcal skin infection sites. Here, we show *h*18:0 stimulates the transcription of a set of lipid metabolism genes associated with the activation of peroxisome proliferator activated receptor (PPAR) in the RAW 264.7 macrophage cell line and mouse primary bone marrow-derived macrophages. Cell-based transcriptional reporter assays show *h*18:0 selectively activates PPARα. Radiolabeling experiments with bone marrow-derived macrophages show [1-^14^C]*h*18:0 is not incorporated into cellular lipids, but is degraded by β-oxidation, and mass spectrometry detected shortened fragments of *h*18:0 released into the media. The catabolism of *h*18:0 was >10-fold lower in bone marrow-derived macrophages isolated from *Ppara* ^−/−^ knockout mice, and we recover 74-fold fewer *S. aureus* cells from the skin infection site of *Ppara* ^−/−^ knockout mice compared to wildtype mice. These data identify PPARα as a target for oleate hydratase-derived hydroxy fatty acids and support the existence of an oleate hydratase-PPARα signaling axis that functions to suppress the innate immune response to *S. aureus*.

## Introduction

Bacterial oleate hydratase (OhyA) activity (EC 4.2.1.53) was first detected in 1962 (Wallen et al., 1962), and the product was characterized as 10(*R*)-hydroxyoctadecanoic acid (*h*18:0) (Mortimer and Niehaus, 1974). OhyA genes are found in bacteria, but not mammals, and encode hydratases that act on mammalian unsaturated fatty acids that contain either a 9*Z* or 12*Z* double bond (Schmid et al., 2016; Demming et al., 2018; Volkov et al., 2010; Kishino et al., 2013). The hydroxy fatty acids are not used by the bacteria but are rather released into the environment (Radka et al., 2021a; Subramanian et al., 2019). Symbiotic bacteria from the intestinal microbiome use OhyA to make these oxygenated metabolites, sometimes as part of a larger pathway for the biotransformation of dietary unsaturated fatty acids to new metabolites (Kishino et al., 2013; Devillard et al., 2007; Salsinha et al., 2018). The best studied hydroxy fatty acid from the intestinal microbiome is 10-hydroxy-12-*cis*-octadecenoic acid (*h*18:1). OhyA products reduce gut inflammation (Miyamoto et al., 2015; Saika et al., 2019), and treatment of animal models with *h*18:1 confers resistance to obesity (Miyamoto et al., 2019), improves the recovery of intestines from dextran sulfate sodium-induced colitis (Miyamoto et al., 2015) and gingival tissue following a periodontal infection (Yamada et al., 2018). These experiments have given rise to the concept that the OhyA products produced by commensal bacteria function to suppress activation of the innate immune system to create a more tolerant environment for the symbionts (Schoeler and Caesar, 2019; Hosomi et al., 2020). Cell models show that OhyA products suppress cytokine formation by immune cells in response to TLR receptor activation (Miyamoto et al., 2015; Ikeguchi et al., 2018; Kaikiri et al., 2017; Yang et al., 2017). The OhyA product, *h*18:1, is slightly more potent than the parent molecule, linoleic acid, in activating both the GPR40 (FFAR1) and GRP120 (FFAR4) G-protein coupled receptors (Miyamoto et al., 2015; Miyamoto et al., 2019) that counter inflammatory activation of neutrophils (GPR40) and macrophages (GPR120) (Miyamoto et al., 2019; Kimura et al., 2020). However, the major OhyA metabolite, *h*18:0, does not stimulate either receptor.

The peroxisome proliferator activated receptors (PPAR) are established regulators of mammalian lipid metabolism and immune cell function (Rakhshandehroo et al., 2010; Bougarne et al., 2018; Christofides et al., 2021; Grabacka et al., 2021; Rigamonti et al., 2008; Iacobazzi et al., 2023). PPARs are ligand-activated transcription factors composed of linked ligand and DNA binding domains, and there are three isoforms; α, β/δ, and γ. Upon activation, PPAR heterodimerizes with the retinoid receptor and the heterodimer transfers to the nucleus to bind DNA sequences containing a PPAR response element within target gene promoters to alter the transcriptional landscape (van Raalte et al., 2004). The PPAR isoforms regulate an overlapping set of target genes involved in peroxisome formation, fatty acid metabolism and β-oxidation. The hydroxylated derivatives of linoleic acid, 9- and 13-hydroxyoctadecadienoic acids (9-HODE, 13-HODE), arising from either enzymatic synthesis (Shureiqi et al., 2003; Wedel et al., 2022) or oxidative damage (Upston et al., 1997) are activating ligands for PPARγ (Itoh et al., 2008; Umeno et al., 2020) and PPARα (Lucarelli et al., 2022; Delerive et al., 2000). PPAR activation leads to enhanced peroxisomal β-oxidation and OhyA products stimulate peroxisome proliferation in cultured cells (Morito et al., 2019). The HODEs attenuate inflammatory processes including leukocyte chemotaxis (Henricks et al., 1991), degranulation of polymorphonuclear leukocytes (van de Velde et al., 1995) and suppress the innate immune response and inflammation in gut epithelia and macrophages (Dubrac and Schmuth, 2011; Delerive et al., 1999). The analysis of PPARα knockout mice show that PPARα is necessary for the maintenance of the intestinal barrier and tolerance toward gut microbiota (Manoharan et al., 2016). Taken together, PPARα and/or PPARγ activation functions to silence the inflammatory response (Bougarne et al., 2018; Christofides et al., 2021; Grabacka et al., 2021; Rigamonti et al., 2008; Iacobazzi et al., 2023), as well as initiate a transcriptional program to support fatty acid catabolism.

*Staphylococcus aureus* is a leading cause of infection worldwide (Chalmers and Wylam, 2020) and World Health Organization antibiotic-resistant global priority pathogen. Here, we show that the major OhyA metabolite produced by *S. aureus*, 10-hydroxyoctadecanoic acid (*h*18:0), stimulates a transcriptional program in macrophages that is driven by the activation of PPARα by *h*18:0. PPARα activation suppresses the immune response and activates peroxisomal β-oxidation to destroy the *h*18:0 signal. These data identify PPARα as a target for OhyA-derived hydroxy fatty acids and support the presence of an OhyA-PPARα signaling axis that suppresses the innate immune response to promote *S. aureus* pathogenesis.

## Materials and methods

### Materials

All chemicals and reagents were reagent grade or better. HPLC-grade solvents, DMEM, and FBS were and obtained from Millipore-Sigma or Fisher unless otherwise indicated. [1-^14^C]oleic acid (18:1; 59 mCi/mmol, 0.1 mCi/ml) and obtained from PerkinElmer (Waltham, MA), 10-hydroxyoctadecanoic acid (*h*18:0) was obtained from AA BLOCKS, INC (San Diego, CA), palmitic acid (16:0), stearic acid (18:0), and 18:1 were obtained from Matreya, LLC (State College, PA), 9-octadecadienoic acid (9-HODE) and 13-octadecadienoic acid (13-HODE) were obtained from Cayman Chemical (Ann Arbor, MI). [1-^14^C]*h*18:0 was prepared using purified oleate hydratase as described previously (Radka et al., 2021b). GW7647, GW0742, and rosiglitazone were provided in the Peroxisome Proliferator-Activated Receptors (PPAR) Panel assay from INDIGO Biosciences, Inc. (State College, PA).

### Cell culture

C57BL/6 (Strain 000664) and *Ppara* ^−/−^ knockout (Strain 008154) mice were obtained from Jackson Laboratory (Farmington, CT). Bone marrow was isolated from 8-10 week old C57BL/6 and *Ppara* ^−/−^ knockout mice by the St. Jude Children’s Research Hospital Animal Resources Center, and macrophages were derived from the bone marrow using the method established by Toda et al. (2021). Cells (4.2 x 10^7^) were seeded in 6-well tissue culture plates and grown in DMEM and 10% fetal bovine serum with 1 ng/ml CSF-1 (ThermoFisher) at 37°C 5% CO_2_. On day 6, bone marrow-derived macrophages (BMDM) were washed with phosphate buffered saline and then changed to media containing 1% dimethyl sulfoxide (DMSO) ± fatty acid treatment and incubated 20-24 h. RAW 264.7 mouse macrophage cells (ATCC number TIB-71) were grown in DMEM plus 10% fetal bovine serum containing 1% DMSO ± fatty acid at 37°C 5% CO_2_.

### RNA sequencing

The RNEasy kit (Qiagen) was used to isolate RNA from BMDM cells incubated 20 h in DMEM 1% DMSO ± 20 μM *h*18:0, and the TRIzol reagent (ThermoFisher) was used to isolate RNA from RAW 264.7 cells incubated 20 h in DMEM 1% DMSO ± 100 μM *h*18:0. RNA was quantified using the Quant-iT RiboGreen RNA assay (ThermoFisher) and quality checked by the 2100 Bioanalyzer RNA 6000 Nano assay (Agilent) or 4200 TapeStation High Sensitivity RNA ScreenTape assay (Agilent) prior to library generation. Libraries were prepared from total RNA with the TruSeq Stranded Total RNA Library Prep Kit according to the manufacturer’s instructions (Illumina, PN 20020599). Libraries were analyzed for insert size distribution using the 2100 BioAnalyzer High Sensitivity kit (Agilent), 4200 TapeStation D1000 ScreenTape assay (Agilent), or 5300 Fragment Analyzer NGS fragment kit (Agilent). Libraries were quantified using the Quant-iT PicoGreen ds DNA assay (ThermoFisher) or by low pass sequencing with a MiSeq nano kit (Illumina). Paired end 100 cycle sequencing was performed on a NovaSeq 6000 (Illumina).

### Gene expression profiling

Read alignment to the M22 release of the GRCm38 mm10 *Mus musculus* reference genome was performed with STAR version 2.7 (Dobin et al., 2013). Gene level quantification was determined using RSEM version 1.3.1 (Li and Dewey, 2011). The top 3000 most variable (and informative) genes from each data set were used to perform principal component analysis using the *prcomp* function, available as part of the standard R distribution. Two filtration steps were taken to prepare data for analysis: only protein coding genes were used/non-coding genes were excluded, and genes with very low expression values were removed. Only genes that passed raw read count > median.library.size/10^6^ (i.e., CPM>0.1) [default cutoff] in the smallest sample-size group were included in pairwise comparisons. Differential gene expression was modeled using the *voom* method, which is available in the *limma* R software package (Law et al., 2014). Differentially expressed genes (DEGs) were defined by *P* value < 0.05 or FDR < 0.05. The top DEGs are defined by the cutoff of FDR < 0.05 and ranked by the change in counts per million in descending order. Gene set enrichment analysis was done using the Reactome signatures from the Molecular Signatures Database (https://www.gsea-msigdb.org/gsea/msigdb) release 7.5.

### PPAR reporter assay

Fatty acids were investigated with commercially available assays for human PPARα, PPARβ/δ, and PPARγ (INDIGO Biosciences, Inc., State College, PA). PPAR assay kits contain proprietary cells, media, control compounds, luminescence detection reagents, and 96-well plate. Assays were performed according to the manufacturer protocols. Briefly, PPAR reporter cells were seeded and incubated 4 h at 37°C 5% CO_2_ in cell recovery medium. The medium was exchanged for compound screening medium containing 0.4% DMSO only or 10 μM fatty acid (16:0, 18:0, 18:1, *h*18:0, 9-HODE, or 13-HODE) in 0.4% DMSO. 10 nM GW7647, 3.3 nM GW0742, or 300 nM rosiglitazone in 0.4% DMSO were used as control agonists for PPARα, PPARβ/δ, and PPARγ respectively. A fatty acid titration using PPARα cells was performed using 0, 0.08, 0.16, 0.31, 0.63, 1.25, 2.5, 5, and 10 μM 16:0, 18:0, 18:1, or *h*18:0. Cells incubated 20 h at 37°C 5% CO_2_, then the medium was removed, the luminescence detection reagents were added, and luminescence was quantified using a BioTek microplate reader (Agilent Technologies, Inc; Santa Clara, CA).

### Metabolic labeling

BMDMs isolated from wildtype C57BL/6 mice were incubated with 1 μM [1-^14^C]18:1 with 9 μM 18:1, or 1 μM [1-^14^C]*h*18:0 with 9 μM *h*18:0 in DMEM 1% DMSO. After overnight incubation at 37°C 5% CO_2_, the cells were harvested and washed twice in 1 ml of 10 mg/ml fatty acid free bovine serum albumin in phosphate-buffered saline, then the lipids were extracted using the Bligh and Dyer method (Bligh and Dyer, 1959) from each experiment. Equal amounts of radioactivity (50,000 cpm/sample) of BMDM lipids labeled with [1-^14^C]18:1 or [1-^14^C]*h*18:0 were spotted on a Silica Gel H plate (Analtech, Inc., Newark, DE) and polar lipids were developed in chloroform:methanol:ammonium hydroxide (60/35/8, v/v/v). Fatty acid methyl esters were prepared from 50,000 cpm of each sample as described previously (Radka et al., 2020), and the entire preparation was spotted on a Silica Gel H plate (Analtech, Inc., Newark, DE) and developed in hexane:ether:acetic acid (80/20/1, v/v/v). The labeled lipids were visualized with a PhosphorImager system.

### Mass spectrometry

BMDMs isolated from C57BL/6 or *Ppara* ^−/−^ knockout mice or RAW 264.7 cells were incubated for 24 h ± 10 μM *h*18:0. The cell culture media was collected and the lipids were extracted using the Bligh and Dyer method (Bligh and Dyer, 1959). The lipid extracts were dried under N_2_ and resuspended in chloroform:methanol (1:1). The fatty acid fraction was isolated using a Discovery DSC-NH_2_ solid-phase extraction column (Supelco, Bellefonte, PA) as described previously (Radka et al., 2020). The free fatty acids were converted to their 3-picolylamide derivatives (Radka et al., 2021a; Li and Franke, 2011) for LC-MS/MS analysis. The amount of each *h*FA was calculated based on the known amount of [U-^13^C]18:1 spiked into the sample at the beginning of the sample preparation (Cambridge Isotopes Labs). Picolylamide-*h*FA were analyzed using a Shimadzu Prominence UFLC attached to a QTrap 4500 equipped with a Turbo V ion source (Sciex). Samples (5 µL) were injected onto an XSelect HSS C18, 2.5 μm, 3.0 x 150 mm column (Waters) at 45°C with a flow rate of 0.4 ml/min. Solvent A was 0.1% formic acid in water, and solvent B was acetonitrile with 0.1% formic acid. The HPLC program was the following: starting solvent mixture of 70% A/30% B; 0 to 5 min, isocratic with 30% B; 5 to 15 min, linear gradient to 100% B; 15 to 23 min, isocratic with 100% B; 23 to 25 min, linear gradient to 30% B; 25 to 30 min, isocratic with 30% B. The QTrap 4500 was operated in the positive mode, and the ion source parameters for the picolylamide-*h*FA multiple reaction monitoring (MRM) parameters were: ion spray voltage, 5,500 V; curtain gas, 15 psi; temperature, 300°C; ion source gas 1, 15 psi; ion source gas 2, 20 psi; declustering potential, 25 V, and a collision energy, 40 V. MRM masses (Q1/Q3) were: picolylamide-*h*12:0, 307.1/109.0; picolylamide-*h*14:0, 335.1/109.0; picolylamide-*h*16:0, 363.1/109.0; picolylamide-*h*18:0, 391.1/109.0, and picolylamide-[U-^13^C]18:1, 391.1/109.0. The system was controlled by the Analyst software (Sciex) and analyzed with MultiQuant™ 3.0.2 software (Sciex).

### Skin/soft tissue (SSTI) infection model

The SSTI thigh infection model used 8-12-week-old C57BL/6 (Jackson Laboratory strain 000664) or C57BL/6 *Ppara* ^-/-^ (Jackson Laboratory strain 008154) male and female mice. The *S. aureus* clinical MRSA strain AH1263 was grown in Luria broth overnight with aeration at 37°C and back diluted in fresh medium to grow to an OD_600 =_ 0.4. AH1263 cells were washed twice in phosphate buffered saline and then resuspended in fresh phosphate buffered saline to obtain an inoculum of ~2.5 – 3.5 x 10^6^ CFU/50 μl. AH1263 cells were introduced by 50 μl intramuscular injections, and 11 infections (C57BL/6 or C57BL/6 *Ppara* ^-/-^) were allowed to proceed for 24 hours. The infected tissue were excised and homogenized in 1 ml of ice-cold phosphate buffered saline and the bacterial burden was determined by serial diluting and selective plating using mannitol salt. For further analysis, the homogenized samples were spun down and the supernatant was removed. The cytokines in the supernatant were analyzed by uncoated TNF-α and uncoated IL-6 ELISA kits. The supernatant was diluted 1:4 in phosphate buffered saline and the IL-6 abundance was measured according to the manufacturer’s instructions for the IL-6 ELISA kit (Invitrogen Cat. No. 88-7064). TNF-α was measured in undiluted supernatant according to the manufacturer’s instructions for the TNF-α ELISA kit (Invitrogen Cat. No. 88-7324).

### Data analysis and statistics

Data analyses were performed using Prism version 9 (GraphPad Software, Boston, MA).

### Ethics statement

All animal experiments were performed with prior review and approval by the St. Jude Institutional Animal Care and Use Committee. All mice were maintained in biosafety level 2 facilities in accordance with IACUC protocol number 566-100442-10/16.

### Data availability

All RNA-seq reads generated and used in this study were deposited in the NCBI Sequence Read Archive under BioProject PRJNA1002616.

## Results

### Transcriptional response of macrophages to *h*18:0

**Figure 1:**
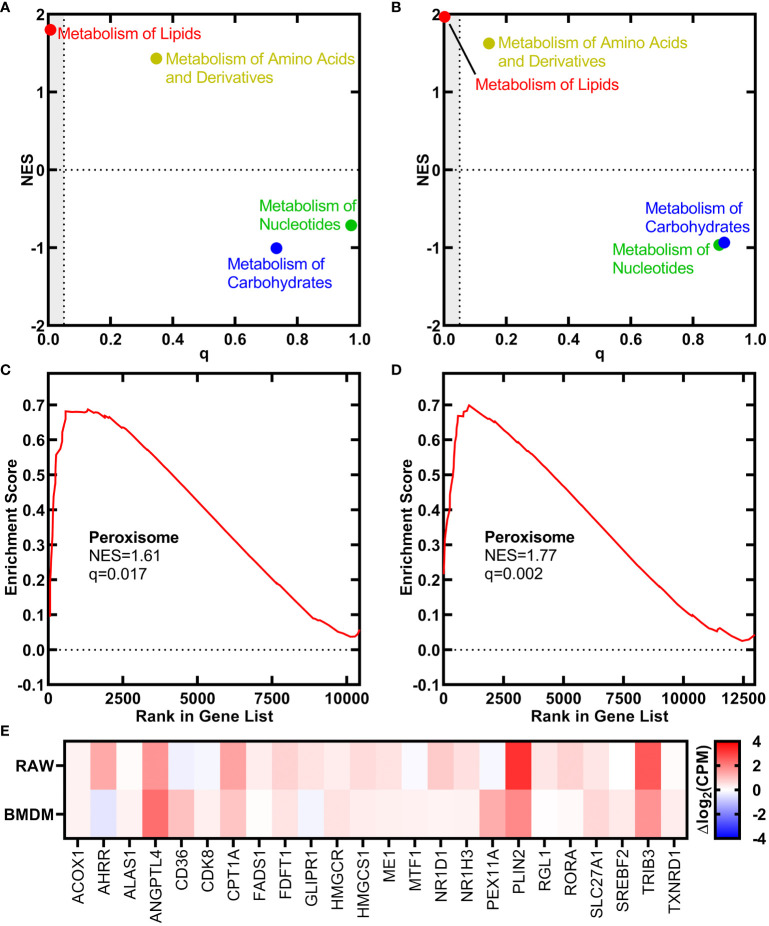
OhyA-derived *h*18:0 initiates a peroxisome proliferation transcriptional program in macrophages. Reactomes from the Molecular Signatures Database (MsigDB, https://www.gsea-msigdb.org/gsea/msigdb) identified by gene set enrichment analysis that are regulated by *h*18:0. Normalized enrichment score (NES) and false discovery rate q-value illustrate the statistical significance for the correlation between the reactome gene set and the gene expression data set. **(A)** RAW cells. **(B)** BMDM. Gene set enrichment analysis for the peroxisome hallmark from MsigDB. **(C)** RAW cells. **(D)** BMDM. **(E)** A heat plot comparison of the regulation of lipid metabolism by PPARα reactome gene set upregulated by *h*18:0 in RAW cells and BMDM.

We used RNAseq to determine if the signaling function of *h*18:0 elicited changes in macrophage gene expression. Both the RAW 264.7 macrophage cell line and primary bone marrow-derived macrophages (BMDM) isolated from C57BL/6 mice were incubated either with or without *h*18:0 for 20 h, the cellular RNA isolated, and RNAseq performed using Illumina NovaSeq. Approximately 90% of the reads mapped to the *Mus musculus* genome in all cases. The reactome classification in the Molecular Signatures Database (MSigDB; https://www.gsea-msigdb.org/gsea/msigdb) for gene set enrichment analysis was used to classify the differentially expressed genes into functional pathways. This analysis showed that genes related to the metabolism of lipids were the most highly up regulated group of genes in the dataset in both RAW cells (**Figure 1A**; q = 0.007) and BMDM (**Figure 1B**; q = 0.002). Genes associated with the proliferation of peroxisomes were significantly up regulated in both the RAW cell line (**Figure 1C**) and BMDM (**Figure 1D**). A heat plot of the salient lipid metabolic genes associated with PPAR regulation in the RAW cell line and BMDM highlight the similarities in the genetic response to *h*18:0 (**Figure 1E**). These data suggest that *h*18:0 activates one of the PPAR transcriptional regulators, although it is not possible to definitively ascribe the changes elicited by *h*18:0 to a specific PPAR isoform because they regulate a highly overlapping set of genes (Bugge and Mandrup, 2010; Zhang et al., 2015).

### *h*18:0 activates PPARα

**Figure 2:**
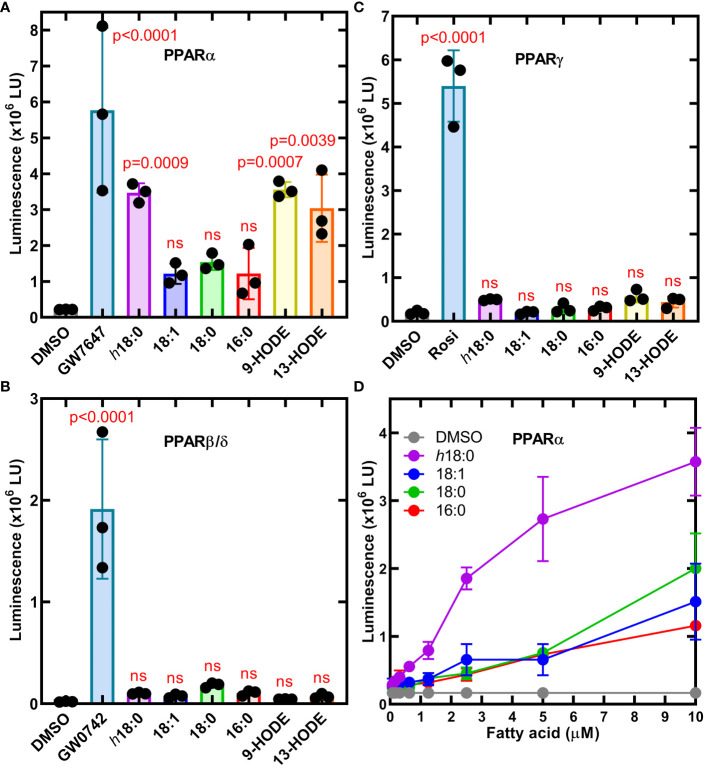
Cellular luminescence assays identify *h*18:0 as a ligand for PPARα. Cell lines were treated with either 0.4% DMSO (control) or 0.4% DMSO containing the indicated ligand and incubated for 20 h and transcriptional activation of the reporter gene was measured by luminescence. One way ANOVA followed by Dunnett’s test was used to compare activation from each ligand to DMSO control. The *P* values are provided for significant differences, and nonsignificant differences are indicated by ns. **(A)** The PPARα reporter line treated with 10 nM GW7647 or 10 μM of the indicated fatty acid. **(B)** The PPARβ/δ reporter line was treated with 3.3 nM GW0742 or 10 μM of the indicated fatty acid. **(C)** PPARγ reporter line stimulated with 300 nM rosiglitazone (Rosi) or 10 μM of the indicated fatty acid. **(D)** Dose-response of the PPARα reporter line to *h*18:0 compared to non-hydroxy fatty acids. Data are Mean ± SD.

We employed a set of three cell lines each with an engineered luminescence reporter gene to detect the ability of ligands to specifically activate each PPAR isoform (Velazquez et al., 2022). Ligands were delivered in DMSO and DMSO alone or with saturating amounts of PPAR-specific ligands that were used as controls to define the assay baseline and the maximal response (**Figure 2**). PPARα was potently activated by *h*18:0 with a response comparable to the maximum signal in the assay (**Figure 2A**). Both 9-HODE and 13-HODE also potently activated PPARα. PPARβ/δ was not activated by any of the hydroxy fatty acids (**Figure 2B**). We measured a modest, insignificant PPARγ response to *h*18:0, 9-HODE and 13-HODE compared to non-hydroxy fatty acids (**Figure 2C**). Although previous reports indicate that PPARγ interacts with 9- and 13-HODE (Itoh et al., 2008; Nagy et al., 1998; Yokoi et al., 2009), in our comparative experiments, the hydroxy fatty acids did not activate PPARγ like they did PPARα. Dose response experiments confirmed the ability of *h*18:0 to activate PPARα-driven transcription (**Figure 2D**). These data point to PPARα as a target for OhyA-derived metabolites.

### Macrophage metabolism of *h*18:0

**Figure 3:**
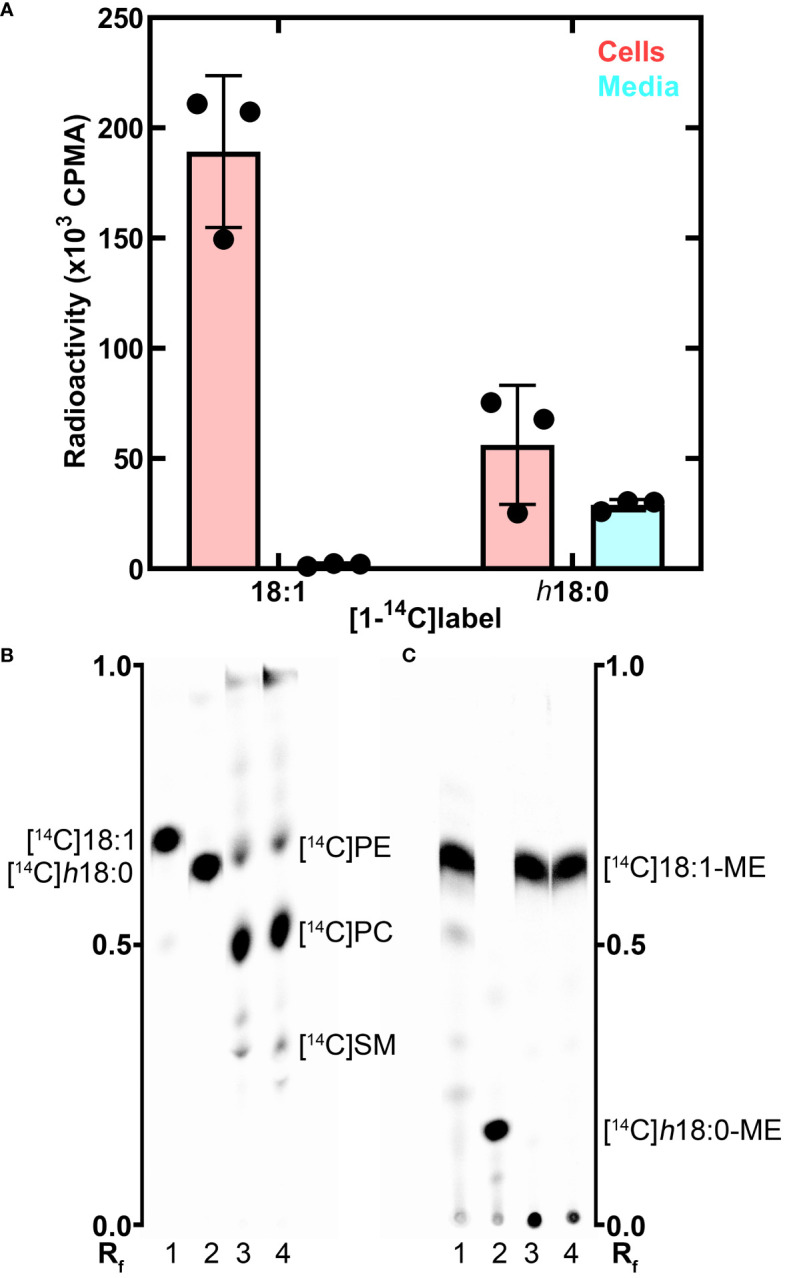
Metabolism of *h*18:0 by BMDM. BMDM were isolated and exposed to either [^14^C]18:1 or [^14^C]*h*18:0, the cells and media separately isolated and the lipids extracted. **(A)** Recovery of radiolabel from the cells and media. **(B)** Separation of the cellular lipids labeled by [^14^C]18:1 or [^14^C]*h*18:0. Equal amounts of radioactivity were separated on Silica Gel H thin layers using chloroform:methanol:ammonium hydroxide (60/35/8, v/v). The locations of the major labeled cellular phospholipids, phosphatidylethanolamine ([^14^C]PE), phosphatidylcholine ([^14^C]PC), and sphingomyelin ([^14^C]SM), are noted. **(C)** The cellular lipids converted to fatty acid methyl esters (ME) and separated on Silica Gel H thin layers using hexane:ether:acetic acid (80/20/1, v/v/v). Lane 1, [^14^C]18:1 control; Lane 2, [^14^C]*h*18:0 control; Lane 3, Lipid extract from BMDM labeled with [^14^C]18:1; Lane 4, Lipid extract from BMDM labeled with [^14^C]*h*18:0. The labeled lipids were visualized with a PhosphorImager system. The R_f_ ruler shows relative migration of each molecule.

The metabolism of *h*18:0 by macrophages was investigated by tracing the fate of radiolabeled [1-^14^C]*h*18:0 prepared enzymatically using *S. aureus* OhyA to hydroxylate [1-^14^C]18:1 (Radka et al., 2021b). BMDM isolated from wildtype C57BL/6 mice were incubated for 24 h with [1-^14^C]18:1 or [1-^14^C]*h*18:0, and the cellular and media lipids were extracted and quantified (**Figure 3A**). Virtually all of the [1-^14^C]18:1 was incorporated into the cells with very little radiolabel remaining in the media. In contrast, the total amount of [1-^14^C]*h*18:0 recovered was less than half of the [1-^14^C]18:1 recovered, and the label was split between the cells and the media indicating incomplete BMDM catabolism of [1-^14^C]*h*18:0 (**Figure 3A**). The lower overall recovery of label derived from [1-^14^C]*h*18:0 indicated that the carbon-14 from *h*18:0 was being converted to non-lipid metabolites.

We followed our bulk analysis of total fatty acid mass balance with an analytical analysis of fatty acid utilization by examining which lipids were labeled using equal counts of radioactivity recovered from the cellular lipids in both sample sets. The distribution of the two labeled fatty acids in the cellular lipids was determined by thin-layer chromatography (**Figure 3B**). Both [1-^14^C]18:1 and [1-^14^C]*h*18:0 labeled the same major cellular phospholipids: phosphatidylcholine, phosphatidylethanolamine and sphingomyelin (**Figure 3B**). Because hydroxy fatty acids are not normally found in mammalian phospholipids, we took the additional step of converting the acyl chains of the cellular lipids to fatty acid methyl esters to determine if [1-^14^C]*h*18:0 was directly incorporated into lipids (**Figure 3C**). The [1-^14^C] atoms from [1-^14^C]18:1 remained as fatty acid as expected. However, the methyl esters derived from [1-^14^C]*h*18:0 were not hydroxylated indicating that these fatty acids were derived from the degradation of [1-^14^C]*h*18:0 to [1-^14^C]acetyl-CoA, which was subsequently incorporated into fatty acids by the macrophage fatty acid biosynthetic pathway. The mass balance of [1-^14^C]*h*18:0 indicates half of the fatty acid disappears from the lipophilic fraction, and the analytical analysis demonstrates *h*18:0 was not used directly for lipid synthesis, but rather degraded and the acetyl-CoA products were recycled to build new acyl chains as shown by the conversion of [1-^14^C]*h*18:0 to [^14^C]18:1.

**Figure 4:**
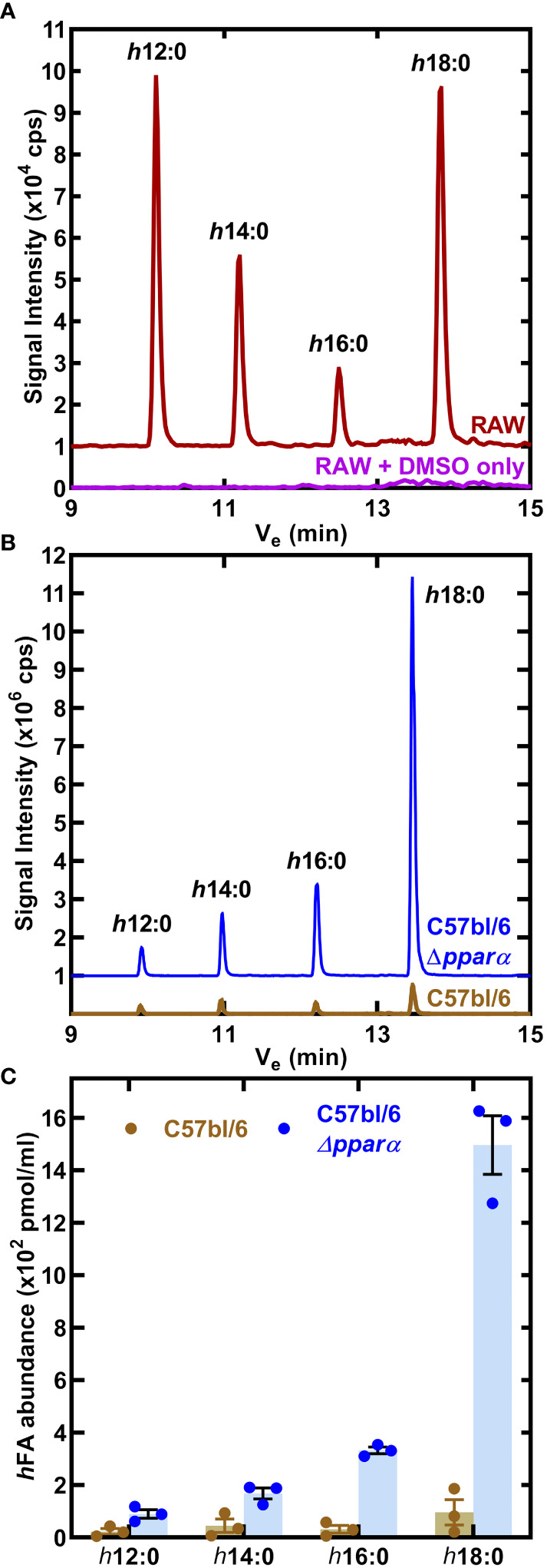
Catabolism of *h*18:0 by macrophages. **(A)** RAW cells were incubated in the presence or absence of 10 μM *h*18:0 for 24 h, media extracted, the fatty acids derivatized and the hydroxy fatty acids profiled by LC-MS/MS. Representative chromatograms of control (DMSO alone) RAW cells compared to RAW cells exposed to *h*18:0. **(B)** Hydroxy fatty acids (*h*FA) recovered from cell culture media following the incubation of BMDM derived from either C57BL/6 or *Ppara^−/−^* mice with *h*18:0. **(C)** Quantification of **(B)** (n=3). Mean ± SD.

RAW 264.7 macrophages were incubated for 24 h with 10 μM *h*18:0, the media was extracted and the fatty acids derivatized to determine the composition of hydroxy fatty acids in the media (Radka et al., 2021a). Neither *h*18:0 nor its shortened products were detected in RAW macrophages confirming that hydroxylated fatty acids were below detection in untreated cultured cells (**Figure 4A**). However, in the presence of *h*18:0, hydroxyhexadecanoic acid (*h*16:0), hydroxytetradecanoic acid (*h*14:0), and hydroxydodecanoic acid (*h*12:0) were detected (**Figure 4A**). These shorter hydroxy fatty acids were interpreted as intermediates in the β-oxidation of *h*18:0 each shortened by one round of β-oxidation.

We determined if the appearance of these truncated hydroxy fatty acids was PPARα dependent by comparing their abundance in BMDM isolated from C57BL/6 or *Ppara* ^−/−^ mice exposed to 10 μM *h*18:0 for 24 h (**Figure 4B**). Media samples were analyzed using LC-MS/MS with [U-^13^C]18:1 as the internal standard to quantify the amounts of hydroxy metabolites present (**Figure 4C**). There was low recovery of *h*18:0 and its metabolites from wild-type BMDM consistent with the robust oxidation of *h*18:0 by primary macrophages. Any modest differences in truncated hydroxy fatty acid distributions are likely related to differences in *h*18:0 catabolism efficiencies between the RAW 264.7 macrophage cell line and primary BMDMs. In contrast, the recovery of *h*18:0 was over an order of magnitude higher in BMDM from *Ppara* ^−/−^ mice indicating that these cells are deficient in the degradation of *h*18:0. The absence of PPARα exacerbates the inefficient catabolism of *h*18:0 (**Figure 3A**) as shown by the accumulation of truncated hydroxy fatty acids (**Figure 4C**). These data are consistent with the activation of PPARα by *h*18:0 activating a PPARα-dependent transcriptional program to accelerate the oxidation and degradation of the *h*18:0 signal.

### Importance of PPARα in promoting *S. aureus* infection

**Figure 5:**
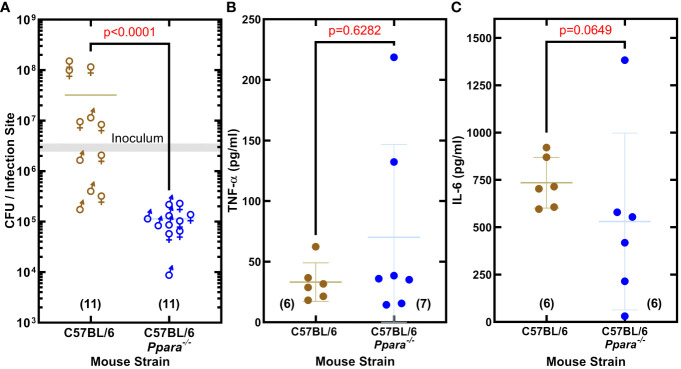
PPARα is an immunological target that promotes *S. aureus* infection. **(A)** Enumeration of the bacteria recovered from the infection site. *S. aureus* clinical MRSA strain AH1263 was used to infect mice by intramuscular infection. The gray shaded bar represents the range of initial inoculum, and the numbers of animals are in parentheses. Mann-Whitney test determined whether overall differences between bacteria recovered from wildtype C57BL/6 or C57BL/6 *Ppara^-/-^* knockout mice infections have statistical significance and determined the two-tailed *P* value. Each data point reflects an infected thigh, and the sex of the animal from which the thigh came is indicated by gender symbol. **(B, C)** Cytokine levels in infected thigh tissue. Error bars show standard deviation of the data. Mann-Whitney tests determined that overall differences in cytokine levels measured in C57BL/6 or C57BL/6 *Ppara^-/-^* knockout mice infections are not statistically significant and determined the two-tailed *P* value. Each data point reflects an infected thigh, and the number of thighs analyzed in each group are indicated in parentheses.

The impact of PPARα on *S. aureus* virulence was assessed in an immunocompetent skin/soft tissue (SSTI) infection model using C57BL/6 and C57BL/6 *Ppara^-/-^* mice. The SSTI infection model showed *S. aureus* established an infection in wildtype C57BL/6 mice, but the bacterial burden in the *Ppara* ^−/−^ mice was 74-fold lower (**Figure 5A**). The same inoculum was used to infect C57BL/6 and *Ppara^-/-^* mice, and the differences in the titers observed could be attributed to both pathogen outgrowth (or lack thereof) as well as innate immune clearance. We measured the abundance of TNF-α and IL-6 in the thigh muscle tissue and found similar levels of both cytokines in C57BL/6 and *Ppara^-/-^* infections (**Figures 5B, C**). C57BL/6 mice have a genetic predisposition that confers greater resistance to *S. aureus* infection in terms of control of bacterial growth and survival compared to other mouse strains (von Kockritz-Blickwede et al., 2008). Enhanced neutrophil recruitment to the infection site is proposed to be a major component of the superior C57BL/6 innate immune response and resistance to staphylococcal infection (von Kockritz-Blickwede et al., 2008). These data show that PPARα signaling is also an important component, playing a key role in promoting *S. aureus* survival in the host and linking bacterial unsaturated fatty acid hydroxylation to the initiation of a specific mammalian signal transduction pathway. The staphylococcal infection phenotype shows the robust impact of PPARα on the innate immune response, even in a resistant mouse strain.

## Discussion

**Figure 6:**
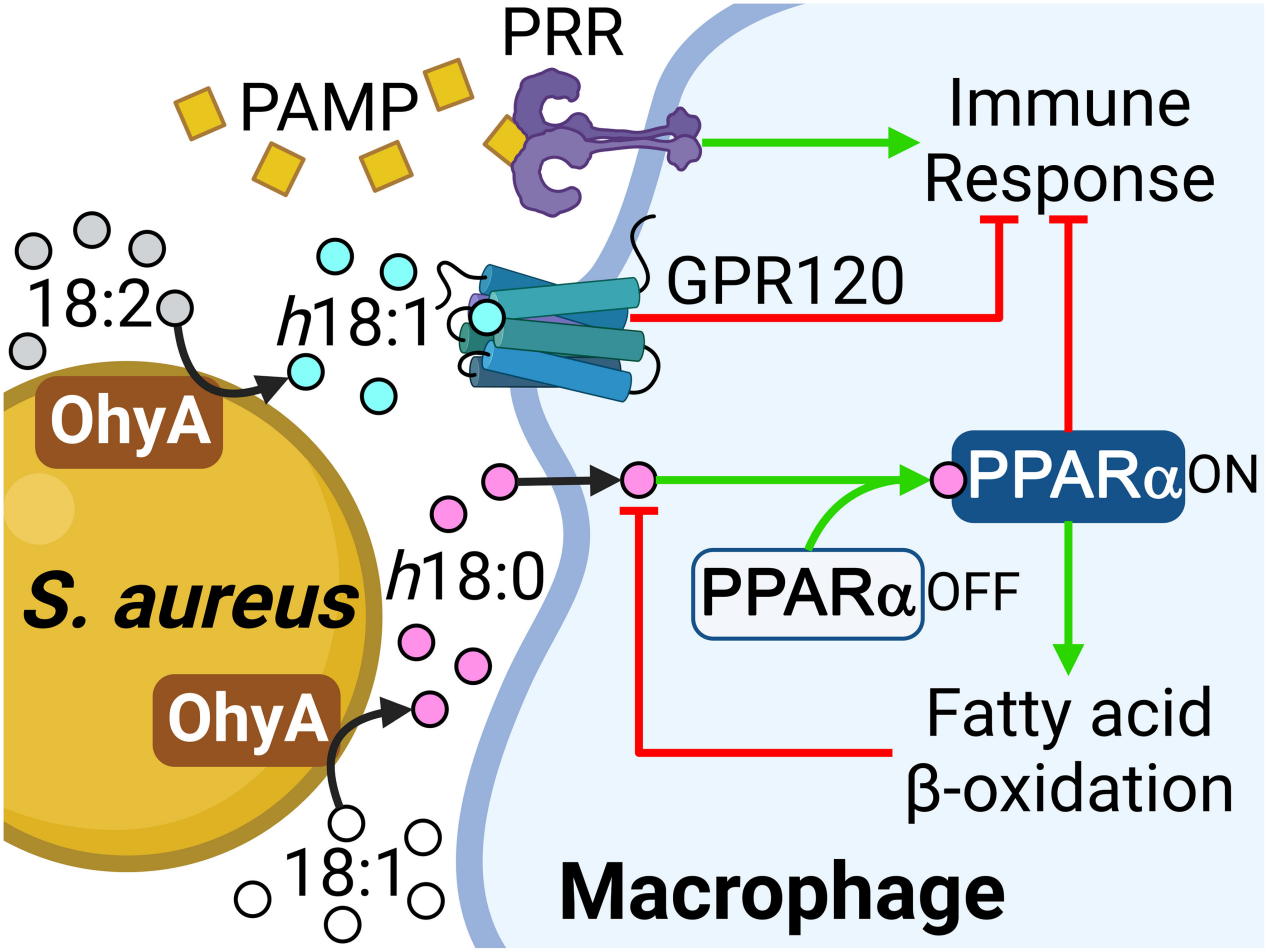
Role of the OhyA-PPARα signaling axis in promoting *S. aureus* pathogenesis. *S. aureus* obtains oleic acid from the host and converts this fatty acid to *h*18:0, which is released into the environment at the infection site. *S. aureus* releases pathogen-associated molecular patterns (PAMP) that trigger the pattern recognition receptors (PRR) on the macrophage surface and stimulate the production of countermeasures to combat the infection. The *h*18:1 released by *S. aureus* is an activating ligand for GPR120. *h*18:0 does not stimulate GPR120 and enters the macrophage to interact with PPARα. Both PPARα and GPR120 activation suppress the innate immune response and PPARα elevates the expression of fatty acid oxidation genes to degrade the hydroxy fatty acid signals. Created with BioRender.com.

The anti-inflammatory suppressive effect of *h*18:0 on nitrite production in RAW 264.7 macrophage cells stimulated with lipopolysaccharide has been shown in cell culture (Yang et al., 2017), but the underlying molecular mechanism(s) responsible for this effect are unclear. Our work establishes the existence of an OhyA-PPARα signaling axis that links the major hydroxy fatty acid produced by *S. aureus* and gut commensal bacteria to the activation of PPARα (**Figure 6**). *S. aureus* does not synthesize OhyA substrate unsaturated fatty acids and must obtain them from the host (Radka et al., 2021a). *S. aureus* OhyA converts the unsaturated fatty acids palmitoleic acid (16:1), oleic acid (18:1), and linoleic acid (18:2) to *h*16:0, *h*18:0, and *h*18:1 hydroxy fatty acids, respectively (Radka et al., 2021a; Subramanian et al., 2019). The hydroxy fatty acids are then released into the environment at the infection site and assist in augmenting bacterial virulence (Radka et al., 2021a). *S. aureus* also releases pathogen-associated molecular patterns (PAMP) that trigger the pattern recognition receptors (PRR) on the macrophage surface (**Figure 6**). The innate immune response to PAMP stimulates the production of countermeasures to combat the infection (Li and Wu, 2021). This response is blunted in macrophages by the activation of GPR120 which attenuates NF-κB signaling via a Tak1-dependent pathway (Oh et al., 2010). *h*18:1 is known to activate GPR120, whereas *h*18:0 does not (Miyamoto et al., 2019). GPR120 activation enhances insulin sensitivity in mice and exerts anti-inflammatory effects in RAW 264.7 macrophage cells and in primary intraperitoneal macrophages (Oh et al., 2010).

The *h*18:0 released by *S. aureus* enters the macrophage and stimulates PPARα that blunts the immune response and initiates a transcriptional program that drives peroxisome proliferation. PPARα stimulation by 9-HODE (**Figure 2A**) suggests *h*18:1 is also likely a PPARα ligand. The first indication that PPARα was involved in immune regulation was the prolonged inflammatory responses observed in *Ppara* ^−/−^ knockout mice (Devchand et al., 1996). PPARα is particularly important to maintain gut homeostasis and tolerance to gut microbiota through suppression of the Th1/Th17 inflammatory response (Manoharan et al., 2016) supporting the connection between the production of OhyA metabolites by commensal bacteria and maintaining mucosal tolerance. Current research is focused on uncovering the molecular basis for the suppression of the innate immune response by PPARα activation (Bougarne et al., 2018; Christofides et al., 2021; Grabacka et al., 2021; Rigamonti et al., 2008; Iacobazzi et al., 2023). Wild-type and Δ*ohyA S. aureus* strains were equally infective in the neutropenic thigh model, but recovery of the Δ*ohyA* strain was 2 orders of magnitude lower in the immunocompetent skin infection model (Radka et al., 2021a). Despite the lower bacterial burden at the infection site, the levels of IL-6, MCP-1, IL-1β and TNF-α elicited by the Δ*ohyA* strain were as robust as either the wild-type or the complemented strain indicating that a more highly activated immune system is responsible for the more effective clearing of the Δ*ohyA* strain. In this study we observed lower bacterial burden at the infection site of *Ppara^-/-^* mice compared to C57BL/6 mice, but the levels IL-6 and TNF-α elicited by *S. aureus* infection were equally robust in both mouse lines. Thus, *S. aureus* has co-opted the OhyA-PPARα signaling axis used by gut commensals to delay and attenuate the immune response to infection.

The second response of macrophages to *h*18:0 is to activate the PPARα-driven acceleration of peroxisomal fatty acid oxidation via a well-characterized transcriptional program (Bougarne et al., 2018; Pawlak et al., 2015). We show PPARα-deficient macrophages are unable to efficiently degrade *h*18:0 revealing that PPARα signaling and increased β-oxidation accelerates the degradation of the *h*18:0 signal. Morita et al. (Morito et al., 2019) report that *h*18:1 is degraded by cultured mammalian cells and that degradation is significantly reduced in Chinese hamster ovary cells that lack peroxisomes consistent with hydroxy fatty acid activation of PPARα. Immunological targets that modulate the innate immune response have been identified for two of the hydroxy fatty acid molecular species that are synthesized by *S. aureus* (*h*18:0 and *h*18:1) (**Figure 6**), but not the *h*16:0 lipid product.

Hydroxy fatty acids are not normal components of mammalian lipids, although they are formed constantly from the enzymatic and non-enzymatic oxidation of unsaturated fatty acids. In *h*18:0, the *R*-stereochemistry of the hydroxyl group is the opposite to the *S*-configuration of the hydroxylated intermediates in mitochondrial β-oxidation (Chu and Schulz, 1985); however, the peroxisomes can handle the degradation of this molecule because the β-oxidation complex of peroxisomes possesses 3-hydroxyacyl-CoA epimerase activity that switches the stereochemistry and allows complete degradation of the molecule (Li et al., 1990). Thus, the activation of *h*18:0 degradation by PPARα-driven peroxisomal β-oxidation functions to turn off the signal (**Figure 6**).

Probiotic intestinal bacteria produce a spectrum of hydroxy fatty acids that are derived from dietary polyunsaturated fatty acids. For instance, *Lactobacillus acidophilus* FA-HY1 hydroxylates the 12-*cis* double bond of linoleic acid to produce 13-hydroxy-9-*cis*-octadecenoic acid (Hirata et al., 2015). *Lactobacillus plantarum* cells produce *h*18:1 from linoleic acid, 10-hydroxy-12-*cis*,15-*cis*-octadecenoic acid from α-linoleic acid and 10-hydroxy-12-*cis*,15-*cis*-octadecenoic acid from γ-linoleic acid (Nanthirudjanar et al., 2015). *L. plantarum* hydroxy fatty acids reduce *SREBP-1c* mRNA expression and LXRα activation, which is the proposed mechanism by which *L. plantarum* suppresses lipogenesis and triacylglycerol accumulation in HepG2 liver cancer cells that express low levels of PPARα (Nanthirudjanar et al., 2015). These studies represent the diversity of lipid products that can be made from dietary fatty acids and potential probiotic effects that could be influenced by diet.

## Data availability statement

The RNA-seq datasets presented in this study can be found in online repositories. The names of the repository/repositories and accession number(s) can be found below: https://www.ncbi.nlm.nih.gov/, PRJNA1002616.

## Ethics statement

The animal study was approved by St. Jude Institutional Animal Care and Use Committee. The study was conducted in accordance with the local legislation and institutional requirements.

## Author contributions

CDR: Formal analysis, Investigation, Methodology, Writing – original draft, Writing – review & editing. MF: Investigation, Writing – review & editing. TS: Investigation, Writing – review & editing. CJ: Investigation, Writing – review & editing. JR: Investigation, Writing – review & editing. COR: Formal analysis, Investigation, Methodology, Writing – review & editing.
